# Fairness, Ethnicity, and COVID-19 Ethics: A Discussion of How the Focus on Fairness in Ethical Guidance During the Pandemic Discriminates Against People From Ethnic Minority Backgrounds

**DOI:** 10.1007/s11673-020-09999-2

**Published:** 2020-08-25

**Authors:** Alexis Paton

**Affiliations:** Aston University, Aston St, Birmingham, B4 7ET UK

**Keywords:** COVID-19, Pandemic ethics, Health inequalities, Ethnic minorities, Social sciences and ethics, Ethical guidelines

## Abstract

Recent weeks have seen an increased focus on the ethical response to the COVID-19 pandemic. Ethics guidance has proliferated across Britain, with ethicists and those with a keen interest in ethics in their professions working to produce advice and support for the National Health Service. The guiding principles of the pandemic have emerged, in one form or another, to favour fairness, especially with regard to allocating resources and prioritizing care. However, fairness is not equivalent to equity when it comes to healthcare, and the focus on fairness means that existing guidance inadvertently discriminates against people from ethnic minority backgrounds. Drawing on early criticisms of existing clinical guidance (for example, the frailty decision tool) and ethical guidance in Britain, this essay will discuss the importance of including sociology, specifically the relationship between ethnicity and health, in any ethical and clinical guidance for care during the pandemic in the United Kingdom. To do otherwise, I will argue, would be actively choosing to allow a proportion of the British population to die for no other reason than their ethnic background. Finally, I will end by arguing why sociology must be a key component in any guidance, outlining how sociology was incorporated into the cross-college guidance produced by the Royal College of Physicians.

## Introduction

Recent weeks have seen an increased focus on the ethical response to the COVID-19 pandemic. Notably absent in Britain, despite calls from several sources (Paton 2020; Fritz, Houlton, and Fuld 2020; Archard and Whittal 2020; Royal College of Physicians 2020), is obligatory national ethics guidance on care during the COVID-19 pandemic.^1^ In its absence, ethics guidance has proliferated, with ethicists and those with a keen interest in ethics in their professions working to produce advice and support for the National Health Service (NHS). The Royal College of Physicians has issued cross-college guidance for all medical frontline staff (2020), and the British Medical Association has issued guidance for doctors (2020). Beyond the professional bodies, the Nuffield Council of Bioethics has issued advice on research and global and government responsibilities during the pandemic.^2^ Internationally, the Hastings Center has issued guidance for the United States, the worst hit country in the pandemic. Despite ethics scholars focusing on slightly different professions and using different theoretical approaches to crafting their guidance, globally there is a consensus that ethics guidance is needed and consensus on what that guidance should look like. The guiding principles of the pandemic have emerged, in one form or another, to favour accountability, transparency, the duty of care, equity, and respect for patients (Royal College of Physicians 2020).

Uniting these principles is the importance of “fairness” in pandemic planning, guidance, and policy. Fairness, whether through a utilitarian approach to assigning limited resources (as seen in Italy) or a reverse triage approach (advocated by the Royal College of Physicians), has emerged as the paramount value of COVID-19 good practice. However, this reliance on fairness is misguided, as the pandemic is categorically unfair. This is because the value of “fairness” when operationalized into guidance, and resulting policy, fails to account for the many inherent ways that our society is not fair when it comes to health. Despite government pronouncements that the pandemic does not discriminate, COVID-19 has in fact disproportionately discriminated against certain groups of British society, hitting people from ethnic minority backgrounds and those living in areas of high deprivation the hardest (Cook et al. 2020; Paton et al. 2020). Fairness does not necessarily mean “equity” when it comes to health. In the rush to create “fair” guidance, the social sciences have been largely forgotten. This has resulted in inadvertent discriminatory guidance, as COVID-19 lays bare what happens when policy and guidance ignore the social determinants of health and illness and the resulting health inequalities they create.

Using evidence on the impact of COVID-19 on people from ethnic minority backgrounds^3^ and drawing on criticisms of existing clinical guidance using sociological theory and evidence, this essay discusses the importance of including sociology in ethical, but also clinical, guidance for care during the pandemic in the United Kingdom. To do otherwise would be actively choosing to allow a proportion of the British population to die for no other reason than their ethnic background. Finally, I show how sociology, as one of the fields that “does” bioethics, is a necessary component for ethics guidance, outlining how sociology has been incorporated into the cross-college guidance produced by the Royal College of Physicians.

## The Impact of COVID-19 on People From Ethnic Minority Backgrounds

Currently 35 per cent of patients critically ill with confirmed COVID-19 are from the Black, Asian, and minority ethnic (BAME)^4^ population in Britain (Intensive Care National Audit and Research Centre [ICNARC] 2020), and an equally disproportionate number of the BAME population have died from the virus (Barr et al. 2020). This is excessively high given that this same group comprises only 14 per cent of the U.K. population (ICNARC 2020). However, these numbers are not just about risk factors related to ethnicity. They raise deeper questions about how ethical and clinical guidance has influenced the pandemic measures taken, measures which have exacerbated the relationship between ethnicity, living conditions, occupation, underlying health conditions, and deprivation. In every instance, the guidance and measures unfairly disadvantage the BAME population.

Take for example the guidelines for social distancing and living conditions. While 79.1 per cent of the white population lives in densely populated urban areas, where it is difficult to practice social distancing, 98.1 per cent of Black and 97.4 per cent of Asian minority groups live in those same environments, making them more susceptible to infection than the white population (ONS 2019). Additionally, the rate of overcrowding for all BAME groups (range 3 per cent to 30 per cent) is higher than in white British households (2 per cent) (Ministry of Housing 2015, 2016, 2017).

People from BAME backgrounds are also over-represented in higher-risk, essential occupations. Doctors from BAME backgrounds represent around 40 per cent of the existing medical workforce in the United Kingdom (Nagpual 2020), with an estimated 64 per cent of COVID-19 related healthcare worker deaths being people from BAME backgrounds (Cook et al. 2020). Beyond healthcare, BAME workers are over-represented in sectors that have continued to work face-to-face, like transport, distribution, and retail sectors (McGregor-Smith 2017).

Besides being an inconvenient truth for the British health and social care system that our current government wishes to ignore, consistent dismissal of the social determinants of health and illness has led to an ignorance of its important role when developing guidance for healthcare. Historically there has been very little policy or public health work done on mitigating ethnic health inequalities, as they have often been wrongly attributed to differences between cultures (Chouhan and Nazroo 2020). However, the need for new guidance in the pandemic provides an opportunity to reorient policy and public health measures such that they consider “the social character of ethnicity, and the socially and economically determined nature of health” (Chouhan and Nazroo 2020, 73). Instead, the focus has been generalizability in an attempt to be “fair.”

## Is Existing Guidance Really Fair?

The focus on generalizability glosses over important ways that health is influenced by ethnicity. Ethics guidance that focuses on generalizability ignores the social scientific evidence that not everyone has an equal chance at good health or even equal access to healthcare services. Advising that it is okay to choose certain patients over others for escalation of care in a pandemic^5^ has trickled down to clinical guidance, where it unknowingly, but actively, discriminates against people from ethnic minority backgrounds. If you are allowed to choose between patients, then criteria are necessary to make that choice. Much of the existing guidance argues that, for fairness, the criteria should as much as possible be about objective, clinical outcomes; however, social outcomes have been discussed too (British Medical Association 2020).

However, existing clinical guidance designed to prioritize patients fairly for escalation of care discriminates against people from ethnic minority backgrounds by using co-morbidities more common in the BAME population to exclude patients. For example, heart disease is one of the most common underlying conditions in deadly cases of COVID-19 (Office for National Statistics 2020). Heart disease has a higher prevalence in three ethnic minority groups well represented in the British population—South Asian, African, and African Caribbean (British Heart Foundation 2020)—thus disproportionately affecting the clinical outcomes of these groups due to ethnicity. Similarly, asthma has a higher prevalence in BAME groups (Netuveli, Hurwitz, and Sheikh 2005; Sheikh et al. 2016), in large part due to their over-representation in more deprived areas with poor air quality (Guarnieri and Balmes 2014; Cai et al. 2017)**.** A concerning fact when asthma is one of the underlying conditions for which individuals must shield themselves for twelve weeks to avoid possible hospitalization should they fall ill (Public Health England 2020).

Failure to work with social scientists and data from the social sciences only serves to worsen the already existing systematic disadvantage that BAME patients already find themselves in with regards to health and well-being (Postnote 2007; Daugherty Biddeson et al. 2019; BMJ 2020; Chouhan and Nazroo 2020). Previous work on pandemic clinical guidance has shown that when emphasis is placed on long-term survival and co-morbidity to determine priority of care, existing clinical guidance (such as the frailty tool) disproportionately disadvantages the BAME population (Daugherty Biddeson et al. 2019). For example, people of South Asian background are at a higher risk of cardiovascular problems, such as angina (British Heart Foundation 2020). The documents that the National Institute of Health and Care Excellence (N.I.C.E.) provides doctors to support decision-making during the pandemic focus on mortality in cardiovascular co-morbidities, explicitly naming angina as a condition that could be used to determine whether escalation to critical care is appropriate for COVID-19 patients (N.I.C.E. 2020). The focus on underlying heart disease to exclude patients from critical care, while clinically relevant, is just one example of how BAME groups will not receive the same care as white British patients if existing guidance fails to take account of the relationship between ethnicity and health.

Additionally, generalizability, in the form of universality, rarely works well when bioethics is put into practice (Paton 2017). Previous work using sociological research has shown that ethical theory developed without empirical data on the context within which it will be used and understood is considered unhelpful by patients and staff in supporting ethical medical practice (Padela et al. 2015; Paton 2017; Paton 2019; Paton and Kotzee 2019). This is not just another shot fired in the empirical versus theoretical debate that rages in bioethics literature^6^ but an important point about ensuring relevancy in the advice that bioethics is supposed to provide. In a pandemic, but in other areas of medicine too, this advice has life or death consequences. Put simply, when the social sciences are ignored in favour of theory developed by white philosophers several hundred years dead, the wrong people die, in their thousands.

As another example, consider how generalizability in ethical guidelines for medical frontline staff actively discriminates against the BAME population. Current ethics guidance focuses on protecting staff with acknowledged clinical vulnerabilities, advocating that these staff be shielded or redeployed to protect themselves from infection. Despite the now established relationship between ethnicity and poor outcomes, there is currently no specific ethics guidance regarding BAME frontline staff who are also potentially vulnerable because of this relationship. As a result, as a group they are not being offered the same options to protect and shield themselves. Given that they make up almost half of our medical workforce, guidance on how best to protect our BAME medical frontline staff during the pandemic is urgently needed but has yet to be developed by the government. Ethics guidance that promotes a duty of care towards clinician well-being, must explicitly advocate that BAME staff be additionally shielded through redeployment or additional personal protective equipment to best protect them from the increased risk of infection and death.

## Building Ethical and Clinical Guidance on a Social Sciences Foundation

Oddly, making guidelines truly fair is relatively easy, though governments have been resistant to these changes. For example, clinical guidance could consider research following the H1N1 pandemic in the United States (Daugherty Biddeson et al. 2019),^7^ which recommends that decisions about long-term survival based on co-morbidities be limited to those patients whose underlying conditions mean they are expected to die in under twelve months. Those with co-morbidities that will live beyond twelve months should not have their co-morbidities used to determine eligibility for treatment, thus helping to avoid discrimination of those groups more likely to have been living with these conditions (often long-term) due to ethnic and/or socio-economic background.

Equally, for ethical guidance we can look to the existing Royal College of Physicians ethical guidance for frontline staff. The Royal College of Physicians took as their starting point an empirical bioethics approach that prioritized sociological work in ethics (Haimes 2002; Scully 2010; Paton 2017, 2018a). Using social science analyses of ethical frameworks and clinical guidance used during the SARS pandemic (Thompson et al. 2006) and the H1N1 pandemic (Daugherty Biddeson et al. 2019), the Royal College of Physicians developed an ethical framework for decision-making based on social scientific work on ethics and pandemics. Reference to this work allowed them to develop with confidence their arguments for accountability, inclusivity, transparency, reasonableness, and responsiveness as guiding principles of the pandemic (Royal College of Physicians 2020), principles known to have been useful during the SARS pandemic of 2003 (Thompson et al. 2006).

Social scientific work on people’s perceptions of a doctor’s duty of care extending to the protection of doctors themselves shaped their advice to frontline staff on working with insufficient personal protective equipment, shielding, and redeployment (Bensimon et al. 2012). Empirical work on the role of cultural perspectives and contexts in pandemic responses (Lor et al. 2016) were used to develop advice for doctors around the importance of considering a patient’s context when making decisions about care priorities. As the Royal College of Physicians guidance shows, it is possible to do things differently and ultimately equitably when developing ethical guidance to best reflect the context within which that guidance will be used.

## Conclusion

Existing ethical and clinical guidelines for care during the pandemic are largely unfair. They actively discriminate against people from ethnic minority backgrounds. When the context within which guidelines will be used are not considered and mitigated by the guidelines themselves, we know more people from ethnic minority backgrounds die for no other reason than a refusal to include evidence from the social sciences during development. In clinical guidance, this is due to a continued blind eye being turned to the social determinants of health and illness and the inherent health inequalities present in the United Kingdom. In ethical guidance, this is due to the continued reliance on older principles, developed without empirical evidence, often by philosophers who could never have conceived of our modern medical era and all the health inequalities contained within that era. Currently endorsed by a majority of faculties and colleges from the Academy of Medical Royal Colleges and the Royal College of Nursing, the Royal College of Physicians guidance shows how taking account of the social sciences in developing ethics guidance develops fair and equitable guidance that advocates against unnecessary and discriminatory deaths during a pandemic.

## Footnotes

1 The Moral and Ethical Advisory Group for the United Kingdom government is using an ethical framework developed in 2007 and updated in 2017. However, this is not a national framework, and it is not obligatory for institutions to adopt it.

2 See https://www.nuffieldbioethics.org/topics/health-and-society/covid-19 for all up-to-date guidance.

3 I could equally have chosen to focus on outcomes of patients from areas of deprivation, but for the sake of brevity, I will focus on only one high-risk group in this article. There is, of course, crossover between these two groups, with many people from ethnic minority backgrounds also living in areas of deprivation, leaving them even more at risk then the equivalent white population.

4 In this article I will use “people from ethnic minority backgrounds” and BAME interchangeably.

5 Such has been done in Italy, a move supported by the BMA in their ethics guidance.

6 One I will not engage in here, as empirical bioethics and the role of sociology in bioethics has been firmly established in the field by Haimes (2002), Scully (2010), and most recently Paton (2017, 2018a, 2018b, 2019).

7 Where pandemics have disproportionately affected minority ethnic groups.
